# Supplementary material for: Association of a Common NOS1AP Variant with Attenuation of QTc Prolongation in Men with Heroin Dependence Undergoing Methadone Treatment
Source: J Pers Med. 2022 May 20;12(5):835. doi: 10.3390/jpm12050835 (PMC9143734; doi:10.3390/jpm12050835)
Supplement: Supplementary file 1 [file jpm-12-00835-s001.zip › jpm-1656828-supplementary.pdf]

Association of a Common *NOS1AP* Variant with Attenuation of QTc Prolongation in Men with Heroin Dependence Undergoing Methadone Treatment

Kuan-Cheng Chang[a, b]* M.D., Ph.D., Ke-Wei Chen[a]*, MD, Chieh-Liang Huang[c]* M.D., Ph.D., Wen-Ling Liao[d, e] Ph.D., Mei-Yao Wu[f, g] M.D., Ph.D, Yu-Kai Lin[a, b] M.D., Yi-Tzone Shiao[h] M.S., Wei-Hsin Chung[a, b] M.D., Yen-Nien Lin[a, b] M.D., Ph.D., and Hsien-Yuan Lane[b, i] M.D., Ph.D.

*K.-C. Chang, K.-W. Chen, and C.-L. Huang contributed equally to the work.

[a]Division of Cardiovascular Medicine, Department of Medicine, China Medical University Hospital, Taichung 404332, TAIWAN

[b]Graduate Institute of Biomedical Sciences, China Medical University, Taichung 404333, TAIWAN

[c]Department of Addiction Treatment, Tsaotun Psychiatric Center, Ministry of Health and Welfare, Nan-Tou County 54249, TAIWAN

[d]Center for Personalized Medicine, China Medical University Hospital, Taichung 404332, TAIWAN

[e]Graduate Institute of Integrated Medicine, China Medical University, Taichung 404333, TAIWAN

[f]School of Post-Baccalaureate Chinese Medicine, China Medical University, Taichung, 40402, TAIWAN

[g]Department of Chinese Medicine, China Medical University Hospital, Taichung 404332, TAIWAN

[h]Center of Institutional Research and Development, Asia University, Taichung 413305 TAIWAN

[i]Department of Psychiatry, China Medical University Hospital, Taichung, 404332, TAIWAN

Supplementary Table S1. Polymerase chain reaction (PCR) primers and probes in the genotyping single-nucleotide polymorphisms (SNPs) spanning the entire *NOS1AP* gene.

Gene	PCR amplification primers	Taq probes
rs1963645-T/C	F: GTG AGT GGG GGC AGG AGA<br>R: AAA CTC CTC TTC AGA AAT TTG AGC	CGA CTG TAG GTG CGT AAC TCT GGG ACG AGG ATG CAG GAA GAT GGA
rs3751284-A/G	F: TGC AGC ACA CGC AGC AGA<br>R: AAG CCG CAG TGC CTA CCT	AGA GCG AGT GAC GCA TAC TAC AGA TGG CCA GGA AGA TGG AGA GAG
rs737641-C/T	F: TAC ACA CTT TCC CTC ACA CAT G<br>R: TTA GCC TTC CTT GGT GCA	GCG GTA GGT TCC CGA CAT ATA CAC AAA ACA CAC ACA CAC ACA CCA
rs164151-T/C	F: TTT ATA TAT ACA TGT GTG AGG GAA AGT G<br>R: AAA ACA CAA ATC TGT TGC TGA TT	GAC CTG GGT GTC GAT ACC TAA GGA TTG TAA CCA GAC GGA AAA GAA
rs1415263-T/C	F: TAA AAT GGA AAT CTG TCA ACT GC<br>R: GTG AGC CCC AAT GTC ACC	AGC GAT CTG CGA GAC CGT ATG GTT GGA AGG CAA CAT ACT TGA TGA
rs945713-C/T	F: AGA GAC TGC AGA AAT GAT AAC TTC A<br>R: ACT TGT ATA TTC TGT GTG ACA CTC C	GGA TGG CGT TCC GTC CTA TTT TTT TGA CAT ATT GAA TTT AAG GTG
rs1572495-C/T	F: TAA TGA AAC AGA GAT CAG GGA CA<br>R: CTG CCT AAA TAT CCT TTG CTT CT	ACG CAC GTC CAC GGT GAT TTA GAG GAT GCA GAT TTG AGC TGA GCC
rs10494366-G/T	F: AAT GAA TTC AAA GGT CAG TTT TAT C<br>R: ATG TGT CCT AGA TAG AGA CCA GTA CA	ACG CAC GTC CAC GGT GAT TTC AGT ACA AAT TCT CAG AAT TTA AAA
rs6683968-T/G	F: TTA AGA CCA TGG TGT CTG G<br>R: AAG TGA AGC TTA GAG AAG TTT AGT AAT CTG	AGC GAT CTG CGA GAC CGT ATT TCA AAG CCT GTC TCT ATA ACT CAC
rs1415257-G/A	F: AAT TAT TCC ACA CAA ACA CCA AA	GCG GTA GGT TCC CGA CAT ATA AGA AAA TCC ACT

Gene	PCR amplification primers	Taq probes
	R: ACT TTT ATA GAG TAA ATT GAA GGG TAT TCA	AGC TGA AAC ACT
rs164148-G/A	F: TAG GCT TTG CCT AGG GAT C	GGC TAT GAT TCG CAA TGC TTT ACT CCT TAA CGG
	R: CAG CCA CTT ATG GCC TTA CA	GCT GGC TGG GGC
rs164149-A/G	F: ATT TTG GGA TAA TCA TAT CCT TGC	AGG GTC TCT ACG CTG ACG ATA GGA TAA AGA CTT
	R: TTA ACA GAT TTT CAA ATG CCT GA	AAT TCC CCG AAC
rs164146-G/C	F: TTG GGT CAG GGA CCA CAG	GTG ATT CTG TAC GTG TCG CCT TGC CAC CCT TGG
	R: AAT TGC ACA TGG CAC TTT TT	CCC ATC TCT CTG
rs2661818-G/C	F: TTA TTT ACT AAA AAC AGC CCA GG	GAC CTG GGT GTC GAT ACC TAG GTC TTC AAC CTC
	R: TGC TAG TGC TCC TGC TCC	CTT CCA CTA TGA
rs164147-C/A	F: ACT GTG AGA CTG TTT TTG TCC AC	AGA TAG AGT CGA TGC CAG CTC TCT GGG TGC GTG
	R: TAG ATC ATA AAA GAT AAG ACA ATT GCA	CCT TGA GCA CAC
rs1876986-A/G	F: TTT GCC TAG GGA TCA CTA CTC C	AGA GCG AGT GAC GCA TAC TAG GGC TGG CTG GGG
	R: TTT GAA AAT CTG TTA ACA GCC AC	CGA TGA GGA AAA
rs1964052-C/T	F: AGC CCT GCT TCC TCT GTG	CGA CTG TAG GTG CGT AAC TCC CAA GAA AGC ACC
	R: TAT GTT TTC TGC TGG AAT ATA GGG	ACC AAA AAC TTA
